# Is the Fixed-Dose Intravenous Trastuzumab Policy Warranted in Limited-Resource Settings?

**DOI:** 10.1200/JGO.18.00155

**Published:** 2019-01-24

**Authors:** Loay Kassem, Kyrillus S. Shohdy, Ahmad M. Abdel-Azeez, Hanaa Attia

**Affiliations:** ^1^ Cairo University Kasr Alainy Faculty of Medicine, Cairo, Egypt; ^2^ Minia University, Minia, Egypt

Trastuzumab (TRA) is a cornerstone treatment for patients with human epidermal growth factor receptor 2–overexpressed breast cancer in adjuvant, neoadjuvant, or metastatic settings. In the pivotal phase III Herceptin Adjuvant trial in patients with early breast cancer, 1 year of TRA was associated with significant survival benefit compared with observation and was found as effective as 2 years of TRA treatment.^1^ Shortage and the unavailability of such essential drugs are pressing issues in low- and middle-income countries. The Egyptian national adjuvant TRA reimbursement program administered by the Egyptian Ministry of Health covers a fixed dose of 440 mg every 3 weeks. These standard regimen adaptations are common scenarios in many countries.^2^ In other countries, local guidelines shorten the 1-year period to a 9-week regimen.^3^

We have reviewed the records of patients with early breast cancer who received TRA as part of their neoadjuvant or adjuvant management in the Clinical Oncology Department of Kasr Alainy School of Medicine during 2 years (2015 and 2016). Eligible patients were those who received at least one dose of TRA for early breast cancer. Loading and maintenance doses, along with the number of cycles and duration of trastuzumab, were extracted for each patient.

One hundred thirteen patients were included in the analysis. Median age was 47 years (range, 22 to 70 years), and 75% commenced treatment with TRA in the adjuvant setting and 25% in the neoadjuvant setting. Baseline characteristics are listed in Table 1. The median number of TRA cycles was nine (range, one to 19 cycles). Mean duration of TRA was 250.6 (± 178.6) days, and mean dose density was every 33.28 (± 15.27) days. Seventy-two patients (63.7%) had dose density of 26 or more days. This was a result of interruptions of the treatment schedule in 87 (77%) patients because of the unavailability of the drug, a delay of reimbursement, or for safety reasons. Among those 87 patients who experienced dose interruptions, only four patients were stopped as a result of cardiotoxicity.

**TABLE 1:**
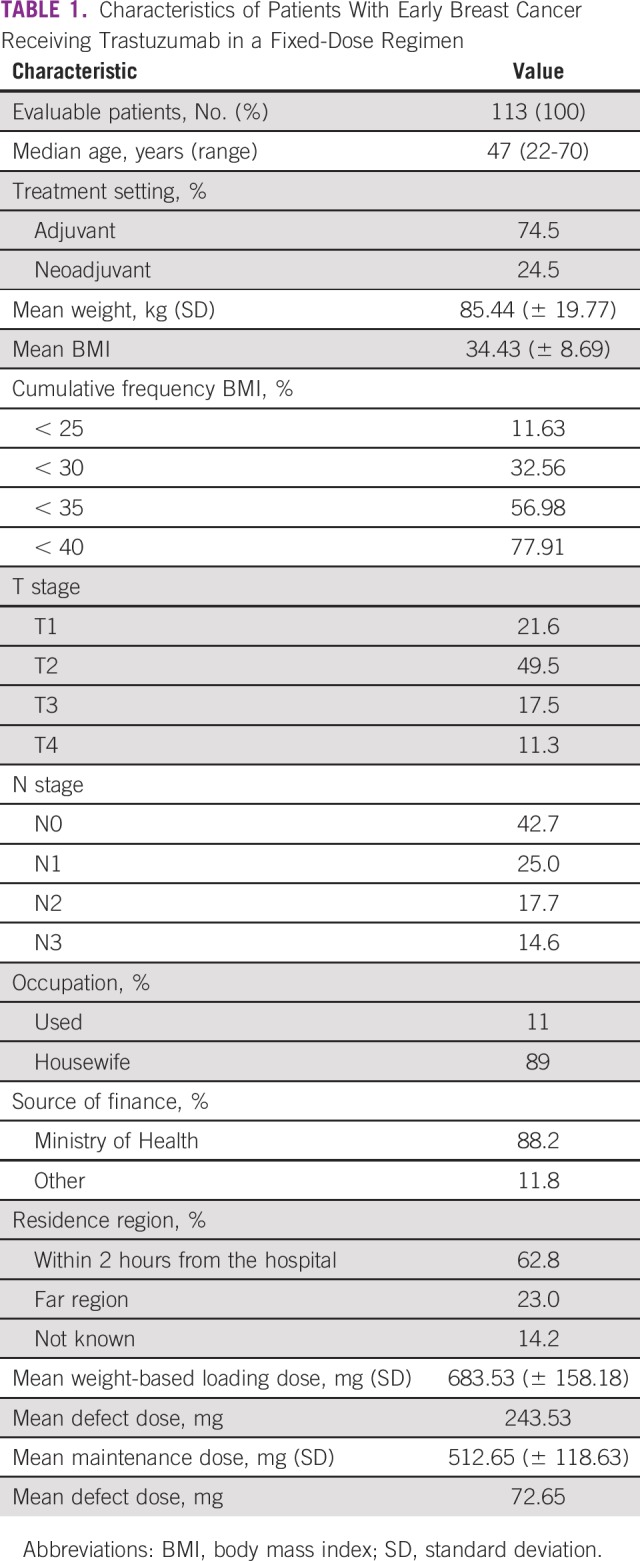
Characteristics of Patients With Early Breast Cancer Receiving Trastuzumab in a Fixed-Dose Regimen

Mean body weight (BW) was 85.44 kg (± 19.77 kg) and mean body mass index (BMI) was 34.43 kg/m^2^ (± 8.69 kg/m^2^), which made 67.44% of the population obese, per WHO definition. This resulted in dosing defects for a significant proportion of patients who received the fixed dose of 440 mg. The drug label recommends a loading dose of TRA 8 mg/kg and a maintenance dose of 6 mg/kg. In our cohort, mean label-recommended (weight-based) loading and maintenance doses were 683.53 mg (± 158.18 mg) and 512.65 mg (± 118.63 mg). Compared with the fixed dose of 440 mg, mean defects in the loading and maintenance doses are 243.53 mg and 72.65 mg, respectively. Rate of underloaded and undermaintained patients (defined by us as patients who need > 500 mg as their loading and maintenance doses, respectively) were 68% and 37.2%, respectively, of the population. Capping the TRA dose in Egyptian patients resulted in a considerable number of patients receiving underloaded and undermaintained doses compared with the weight-based regimen.

Currently, there are no prospective studies evaluating the clinical efficacy of fixed-dose intravenous regimens. One retrospective study from Taiwan^4^ compared the efficacy and safety of weight-based and fixed-dose regimens. Data from 181 patients who received regular weight-based TRA every 3 weeks were compared with that from 119 patients who received monthly fixed doses of 440 mg every 4 weeks as part of adjuvant or palliative treatment. Baseline characteristics were similar in both groups with the exception that the group receiving medication every 4 weeks had a younger population. There was no significant survival difference between the two groups. As expected, median progression-free survival and overall survival were not reached in the adjuvant cohort (*P* = .30 and *P* = .61, respectively). Of interest, on additional analysis using a Cox proportional hazards regression model, the group treated every 4 weeks experienced better progression-free survival than did the group treated every 3 weeks (hazard ratio, 2.445; 95% CI, 1.021 to 5.858; *P* = .045); however, this might be because the group treated every 3 weeks had a higher proportion of patients with stage IIIA to IIIC disease (31.1% *v* 18.6%).

To further evaluate the safety and efficacy of fixed intravenous TRA dosing, two questions must be addressed. First, would discrepancies in patients’ body weight significantly alter the pharmacokinetics (PK) of TRA? Second, will such discrepancies alter the clinical outcome?

As easy to answer as the first question might seem to be, the answer is more complicated. In theory, with the high variability of body weight (and as reported by Wang et al^5^ in comparing the two dosing strategies) fixed doses are expected to overdose patients with low BW and underdose those with high BW. In contrast, theoretically, weight-adjusted dosing can overdose those with high BW and underdose those with low BW. In the same study, and particularly for TRA, similar PK parameters, with regard to simulated areas under the curve and maximum serum concentration variability, were observed for the two dosing schedules.

For the second question, preclinical models established 20 µg/mL as the minimum TRA concentration (C_min_) that achieved maximum tumor growth inhibition.^6^ Although no studies exist on the PK of the intravenous fixed-dose regimen, one study of interest^7^ investigated the PK of a subcutaneous fixed dose of 600 mg in 19 patients with nonmetastatic human epidermal growth factor receptor 2–positive breast cancer. More than one half of the patients did not reach the minimum plasma concentration threshold after the first administration. Moreover, there was an inverse relationship between BMI and plasma concentration of TRA. All patients with BW of 80 kg or more did not exceed the C_min_ threshold. Of note, in this cohort, mean weight was 75.9 kg (± 12.9 kg) and in our cohort even higher (85.44 kg [± 19.77 kg]). This detrimental effect of weight on the pharmacodynamic exposure of the drug was not shown to be clinically significant in the large prospective HannaH (Clinicaltrials.gov identifier: NCT00950300) trial that investigated the PK of subcutaneous TRA.^8^ On subgroup analysis, pathologic complete response rates were equal between the fixed-dose subcutaneous TRA and weight-adjusted intravenous regimens, even in patients with BW greater than 100 kg.^8^

Finally, we acknowledge the difficulty of conducting head-to-head efficacy comparisons to assess the noninferiority of fixed-dose versus the widely used weight-adjusted dosing; however, in our opinion, a three-step plan can clarify this mystery. The first step should compare different PK parameters (as maximum serum concentration, C_min_, and C_trough_) among patients with high versus low BMI who, for financial or other reasons, are offered fixed intravenous dosing. The second step should compare surrogate clinical end points (as pathologic complete response rates and response rates) across different BMI categories in patients who received fixed-dose and weight-adjusted dosing aimed at developing a PK model for the safest fixed dose with the least variability according to BW. Finally, a relatively small prospective PK-oriented study can validate the suggested fixed dose in a manner similar to that used with newer anti–programmed death-1 monoclonal antibodies nivolumab and pembrolizumab.^9^

In conclusion, adopting a fixed 440-mg one-vial dose for every patient is a questionable approach that needs additional assessment. Investing in clinical studies with PK surrogate end points is important in low-resource settings.
